# Evaluating the Performance of Large Language Models in Vascular Surgery Cases: Cross-Sectional Study

**DOI:** 10.2196/91973

**Published:** 2026-11-06

**Authors:** Asanka Wijetunga, Yunyi Wang, Chantell Yaghi, Mauro Vicaretti

**Affiliations:** 1 Westmead Hospital, Cnr Darcy and Hawkesbury Road, Westmead, New South Wales, 2145, Australia, 61 8890 5555 ext 9; 2 The University of Sydney, Sydney, New South Wales, Australia

**Keywords:** surgery, aorta, ischemia, stroke, diabetic foot, artificial intelligence, AI, large language model

## Abstract

**Background:**

Large language models (LLMs) such as ChatGPT, Gemini, and Claude are increasingly used by clinicians, yet their accuracy, safety, and consistency in clinical cases remain poorly defined. Most studies assess LLMs using multiple-choice questions rather than free-response reasoning.

**Objective:**

The primary objective of this study was to evaluate the performance and safety of 3 widely used LLMs in realistic vascular surgery scenarios in key domains such as diagnosis, investigation, and multidisciplinary and multimodal management. The secondary objective of this study was to evaluate the safety of the models’ outputs.

**Methods:**

A total of 42 fictitious vascular cases across 5 major pathologies (acute limb ischemia [ALI], aortopathy, chronic limb ischemia, diabetic foot infection, and extracranial cerebrovascular disease) were independently entered into GPT-5, Gemini 2.5, and Claude Sonnet 4.5 using standardized prompts. Each model answered structured questions covering diagnosis, investigation, and management (both operative and nonoperative). Responses were scored out of 20 by a panel using a predefined rubric. Each plan’s overall safety was separately assessed. Comparative analyses used 2-tailed *t* tests, ANOVA, and multivariable logistic regression.

**Results:**

Mean composite scores by model were 86.5% for ChatGPT, 83.5% for Gemini, and 88.0% for Claude, whereas scores by disease were 82.7% for ALI, 83.8% for aortopathy, 85.9% for chronic limb ischemia, 86.1% for diabetic foot infection, and 80.7% for extracranial cerebrovascular disease (*P*>.05 in each case). Unsafe plans were generated in 11.9%, 23.8%, and 7.1% of cases by ChatGPT, Gemini, and Claude, respectively. On multivariable analysis, independent predictors of unsafe outputs were lower composite score (odds ratio [OR] 0.471, 95% CI 0.306‐0.722; *P*=.001), higher word count (OR 1.003, 95% CI 1.001‐1.005; *P*=.001), and ALI (OR 20.9, 95% CI 3.79-61.8; *P*<.001).

**Conclusions:**

Our findings demonstrate LLMs’ promise in managing routine vascular surgery cases. However, their inconsistent safety profiles and ethical limitations preclude unsupervised clinical use. Rigorous specialty-specific validation is essential before they may be integrated into routine practice.

## Introduction

For many years, clinicians have been using internet search engines such as Google to assist in clinical decision-making [1-3]. However, these tools are imperfect: they do not provide targeted answers to specific questions, retrieved articles can be variable in quality and accuracy, and the order of the search results does not necessarily correlate with their relevance to the given query—with paid advertisements often being given precedence [4,5].

Artificial intelligence (AI)–driven large language models (LLMs) such as OpenAI’s ChatGPT, Google Gemini, and Anthropic’s Claude appear to address many of these shortcomings given their ability to provide rapid, succinct, and targeted responses to specific questions. Since LLMs entered the public domain, many clinicians have begun using these AI tools in numerous ways, including as decision aids in clinical cases, in designing educational material for patients and trainees, and in recordkeeping [6].

These LLMs are powered by a type of neural network known as a transformer, which formulates a response to a query by recognizing patterns in its training database. Numerous potential benefits of LLM-augmented surgical practice have been espoused, including its ability to provide interdisciplinary, unbiased, efficient, and accessible care [7-10]. However, it is too soon to take LLM outputs as fact. LLMs are classed as a “black box technology” as their training data are not publicly disclosed and their outputs lack interpretability or explainability [11]. Furthermore, commercially available LLMs have even been found to confabulate or omit key details in 1% to 5% of responses [11,12].

Despite the uptake of these tools in clinical settings, there is a noteworthy absence of validation studies assessing their performance in vascular surgery scenarios. Many clinicians, especially those who are more junior, are informally “consulting” LLMs by entering fragments of cases to seek management advice. A recent systematic review appraising the role of LLMs in clinical medicine cites only 1 study in vascular surgery out of 156 papers [13] (a small study limited to venous insufficiency [14]), highlighting the evidence gap in this field. Current research has focused on LLM performance in multiple-choice questions rather than free-text clinical reasoning [13,14]. Consequently, the accuracy, safety, and clinical reliability of LLMs when applied to open-ended vascular cases remain largely unexamined.

The aim of this study was to assess the performance of 3 of the most widely used LLMs (GPT-5, Gemini 2.5, and Claude Sonnet 4.5) in each aspect of diagnosis, investigation, and management of routine vascular surgery cases. This will allow clinicians to be better positioned to integrate LLMs into clinical practice with confidence, with improved awareness of common limitations and potential sources of error when evaluating LLM-generated outputs.

## Methods

### Study Design

This was a cross-sectional study evaluating the performance of LLMs in the workup and management of clinical vascular surgery cases. This study compared GPT-5, Gemini 2.5, and Claude Sonnet 4.5 using identical clinical case prompts.

### Case Selection and Design

A total of 42 fictional adult vascular surgery cases in five key pathologies were developed that, given ethical concerns, were not based on real patients and were not generated by the LLMs themselves: (1) acute limb ischemia (ALI), (2) aortopathy, (3) chronic limb ischemia (CLI), (4) diabetic foot infection (DFI), and (5) extracranial cerebrovascular disease (ECD).

Each case was written by a panel of 2 consultant vascular surgeons and 2 vascular surgery trainees. Cases were written in a standardized format including the following variables, with a sample case provided in Multimedia Appendix 1: age; sex; history of presenting concern; medical history; regular medications; surgical history; allergies; social history and functional status; vital signs on admission; examination findings; and blood results on admission, including hemoglobin (g/L), white blood cell count (×10^9^/L), estimated glomerular filtration rate (mg/min/1.73 m^2^), C-reactive protein (mg/L), lactate (mmol/L), hemoglobin A_1c_ (percentage), random blood sugar level (mmol/L), and sodium (mmol/L).

### Prompt Engineering and Scoring

Each case was entered as mentioned above into each LLM using a newly created subscription account, creating a new chat each time to prevent memory with respect to the default settings and temperature parameters used in each instance. The LLMs were accessed via their web interface using Google Chrome (version 138) between June 2025 and August 2025 with the default LLM settings. Replication would require the use of the same model names, default settings, and single-prompt entry without iterative refinement. Each case was entered once per model, and intramodel variability due to stochastic outputs was not assessed.

Each case was introduced with the following standardized prompt: “You are a vascular surgeon, and you have been asked to review the following patient in the emergency department. Please review the case and answer the following questions.” The following 11 questions were asked in each case, and each question was assigned a score (the maximum possible score for each question is listed in parentheses). Responses were collected in full-text format without editing or reprompting. By summing the scores for each question, composite scores out of 20 were generated for each case:

1. What is the most likely diagnosis? (1)
2. What further investigation is required? (3)
3. Does this patient require admission to hospital? (1)
4. Does this patient require urgent surgery during this admission? (1)
5. Does this patient require elective surgery as an outpatient? (1)
6. What operation would be required (if either question 4 or 5 was answered as “yes”)? (3)
7. What pharmacological therapies are required? (2)
8. If this patient required surgery, which medications must be withheld? (1)
9. What nonpharmacological therapies are required? (3)
10. Which other medical specialists are required for this patient’s management? (3)
11. Which allied health specialists are required for this patient’s management? (1)

At the commencement of the study, a marking crib sheet was created by the authors for each case based on contemporary international clinical practice guidelines supplemented by unit-level consensus (Multimedia Appendix 2). Where acceptable practice variation existed (eg, endovascular vs open preference in anatomically suitable patients), multiple approaches were considered correct if supported by guideline-based reasoning. To ensure consistency, a pilot study that involved discussion of the rubric with a small number of cases was presented at an international vascular surgery meeting in August 2025, with further rubric refinement performed prior to final case analysis [15].

The responses were evaluated by the 4-member panel against the predefined scoring rubric in a consensus-based format. Independent blinded scoring was not performed. Safety flags raised by any panel member were discussed collectively; if disagreement persisted, an independent vascular surgeon was available to adjudicate, although this was not required in practice.

A consensus approach was chosen over independent blinded scoring for 2 key reasons. First, it mirrors our unit’s practice, where complex cases are discussed at a departmental level, with a treatment consensus being reached following active collaborative deliberation. Second, the aim of our study was to benchmark outputs against the unit-level standard of care rather than against individual surgeons; hence, this approach would better address the study’s aims.

In addition to the overall score, the overall safety of each plan was assessed. Plans were marked as unsafe if they contained any of the following recommendations that, if acted upon, would reasonably result in serious patient harm (death, avoidable morbidity requiring additional surgical intervention, avoidable long-term disability such as limb loss, avoidable intensive care admission, or avoidable prolonged hospitalization): diagnostic error or delay, delayed or inappropriate surgical intervention, inappropriate prescription or withholding of pharmacotherapy, inappropriate adjunctive therapy, and unsafe disposition.

An output was classified as unsafe if any single element satisfied the aforementioned criteria irrespective of the integrity of the remaining plan. If there was discord within the panel regarding the safety of the outputs, a third vascular surgeon from the unit was on call for independent comment. However, this surgeon was not called upon as assent was reached in each situation flagged as unsafe by a panel member. Any additional suggestions that were not part of our marking crib were reviewed by the panel. If deemed beneficial to patient care, they were noted as additional suggestions. The word count for each response was assessed using Readability Pro (Vercel AI).

### Statistical Analysis

Descriptive statistics were generated for each domain and overall performance. Continuous variables were presented as means and SDs, and categorical outcomes were presented as proportions. Differences were analyzed using one-way ANOVA or independent-sample *t* tests for continuous outcomes as required and chi-square tests for categorical variables. Significance was defined as a *P* value <.05 (2 tailed). Multivariable logistic regression was performed with unsafe output (yes or no) as the dependent variable at the level of individual model case responses (n=126). Variables were selected a priori based on clinical plausibility and univariable significance (*P*<.10). These included composite score (continuous), word count (continuous and per word), and pathology category (ALI vs non-ALI). Collinearity was not formally assessed. Odds ratios (ORs) with 95% CIs were calculated. All analyses were performed using SPSS (version 29.0.0.2; IBM Corp).

### Ethical Considerations

No human or identifiable patient data were used. All cases were fictitious and created for research purposes. This study adhered to the ethical principles of the Declaration of Helsinki. Ethics approval was deemed unnecessary for this study by the Western Sydney Local Health District Research Ethics and Governance Office (submission case number 2025/PID00407), as no human subjects were involved.

## Results

A total of 42 vascular case vignettes were created de novo and then studied across the 3 LLMs. A balanced variety of pathologies were included: 5 (11.9%) cases of ALI, 10 (23.8%) cases of aortopathy, 7 (16.7%) cases of CLI, 10 (23.8%) cases of DFI, and 10 (23.8%) cases of ECD.

### Performance by Model

Mean composite scores by model were 86.5% for GPT-5, 83.5% for Gemini, and 88.0% for Claude, whereas scores by disease were 82.7% for ALI, 83.8% for aortopathy, 85.9% for CLI, 86.1% for DFI, and 80.7% for ECD (*P*>.05 in each case; Multimedia Appendix 3). Unsafe plans occurred were generated in 11.9%, 23.8%, and 7.1% of cases by GPT-5, Gemini, and Claude, respectively.

Composite performance did not substantially vary between models (Table 1). Claude achieved the highest overall score (mean 17.2, SD 1.7), followed by ChatGPT (mean 16.9, SD 1.9) and Gemini (mean 16.2, SD 2.8). There was no significant difference between the models’ overall scores (Supplementary Table S1 in Multimedia Appendix 4). Score ranges were broadest for Gemini (5-20) and narrowest for Claude (12-20). Across individual diseases, variation was minimal, with models scoring comparably across all pathologies, except in the case of ALI (mean 15.0, SD 3.1 to mean 18.2, SD 1.8), where variation was over 10%. Claude significantly outperformed Gemini in both ALI (mean 15.0, SD 3.1 vs mean 18.2, SD 1.8; *P*=.04) and ECD (mean 16.7, SD 1.4 vs mean 15.4, SD 1.3; *P*=.04), and ChatGPT significantly outperformed Gemini in ECD (mean 16.3, SD 0.7 vs mean 15.4, SD 1.3; *P*=.046; Supplementary Table S3 in Multimedia Appendix 4). Gemini generated substantially longer responses (mean 951, SD 316 words) than ChatGPT (mean 664, SD 134 words; *P*=.03) and Claude (mean 715, SD 199 words; *P*=.04; Supplementary Table S1 in Multimedia Appendix 4).

**Table 1.** Scores by model.

Model	Overall score (0-20; n=42 case vignettes), mean (SD; range)	ALI^a^ score (0-20; n=5 case vignettes), mean (SD)	Aortopathy score (0-20; n=10 case vignettes), mean (SD)	CLI^b^ score (0-20; n=7 case vignettes), mean (SD)	DFI^c^ score (0-20; n=10 case vignettes), mean (SD)	ECD^d^ score (0-20; n=10 case vignettes), mean (SD)	Extra suggestions (%)	Unsafe plans (%)	Word count, mean (SD)
ChatGPT	16.9 (1.9; 13-20)	16.4 (2.7)	16.8 (2.0)	17.8 (2.5)	17.1 (1.7)	16.3 (0.7)	56.1	11.9	664 (134)
Gemini	16.2 (2.8; 5-20)	15.0 (3.1)	16.2 (4.1)	17.2 (2.2)	16.8 (2.4)	15.4 (1.3)	57.1	23.8	951 (316)
Claude	17.2 (1.7; 12-20)	18.2 (1.8)	17.2 (2.1)	16.5 (1.6)	17.7 (1.7)	16.7 (1.4)	71.4	7.1	715 (199)

a ALI: acute limb ischemia.

b CLI: chronic limb ischemia.

c DFI: diabetic foot infection.

d ECD: extracranial cerebrovascular disease.

Unsafe plan generation also varied, with Gemini producing the highest proportion of unsafe outputs (23.8%), followed by ChatGPT (11.9%) and Claude (7.1%; Table 1). Although Gemini tended toward more unsafe plans than ChatGPT and Claude, these differences did not reach statistical significance (*P*=.08 and *P*=.06, respectively; (Supplementary Table S1 in Multimedia Appendix 4). Examples of unsafe suggestions can be found in Table 2; they are categorized in Supplementary Table S2 in Multimedia Appendix 4 and categorized by model and disease in Multimedia Appendix 5. Propensity to propose “extra” management steps differed numerically, but not statistically, between models, with Claude suggesting additional measures most frequently (71.4%) compared with ChatGPT (56.1%) and Gemini (57.1%; Textbox 1).

**Table 2.** Suggestions flagged as unsafe, with the number of times they appeared in the sample.

Condition	Unsafe suggestion
ALI^a^ (n=5 case vignettes)	Active limb rewarming offered (n=6) Fasciotomy not offered (n=1) Anticoagulation omitted (n=1) Failure to treat popliteal aneurysm (n=1) Failure to appropriately palliate (n=1)
Aortopathy (n=10 case vignettes)	Advocating for aggressive volume resuscitation for a ruptured aortic aneurysm (n=1)
CLI^b^ (n=7 case vignettes)	Advocating for 6 weeks of bed rest following lower limb bypass surgery (n=1) Recommending outpatient management of a patient with wet ischemic gangrene (n=1)
DFI^c^ (n=10 case vignettes)	Missed diagnosis of necrotizing fasciitis; hence, advocating for the incorrect operation (n=1) Withholding of all anticoagulation after major limb amputation for 5 days in a patient with a metallic aortic valve (n=1) Intensive care not recommended for a patient requiring blood pressure support (n=1)
ECD^d^ (n=10 case vignettes)	Ceasing all antiplatelets in patients following acute ischemic stroke while awaiting endarterectomy (n=4) Recommending endarterectomy as first-line therapy for symptomatic extracranial vertebral artery disease (n=1) Discharge immediately after major stroke (n=1)

a ALI: acute limb ischemia.

b CLI: chronic limb ischemia.

c DFI: diabetic foot infection.

d ECD: extracranial cerebrovascular disease.

Textbox 1. **Examples of additional suggestions proposed by the large language models.**

- Involvement of drug and alcohol specialists in assisting with smoking cessation
- Commencement of an alcohol withdrawal scale and oral thiamine replacement upon admission of a patient with moderate alcohol intake at home
- Involvement of a hand therapist following an acutely ischemic arm
- Dietetics and nutritional prehabilitation prior to elective aortic surgery
- Involvement of inpatient dietetics in a nonhealing ischemic foot wound
- Bariatric surgery referral upon discharge for a patient with obesity after endovascular aortic repair
- Screening for obstructive sleep apnea
- Screening for coronary artery disease using a computed tomography coronary angiogram upon discharge of a patient with chronic limb ischemia

Model-level performance for each clinical domain is summarized in Supplementary Table S4 in Multimedia Appendix 4. There were no statistically significant differences between the LLMs in diagnosis, investigation, admission, or surgical decision-making (*F* range 0.01‐1.74; *P*>.05). Claude demonstrated superior performance in nonpharmacological management (mean composite score, 90.5%; *P*=.01; η^2^=0.06) and consults (mean composite score, 92.9%; *P*=.04; η^2^=0.06), although effect sizes were small.

### Performance by Disease

Scores by disease can be found in Table 3. Although scores between diseases were quite consistent, with ALI obtaining a mean composite score of 16.5 (SD 2.7; 82.5%), aortopathy obtaining a mean composite score of 16.8 (SD 2.9; 84.0%), CLI obtaining a mean composite score of 17.2 (SD 2.1; 86.0%), DFI obtaining a mean composite score of 17.2 (SD 1.9; 86.0%), and ECD obtaining a mean composite score of 16.1 (SD 1.3; 80.5%), minor variations in overall performance were noted. Cases of CLI and DFI scored most highly, significantly outperforming ECD (*P*=.03 and *P*=.01, respectively; Supplementary Table S6 in Multimedia Appendix 4). Separately analyzing each metric using ANOVA demonstrated variability in the LLMs’ abilities to manage each disease (Supplementary Table S5 in Multimedia Appendix 4). The models performed most variably in diagnosis (*P*<.001; with ALI being a significant outlier: 46.7% vs 76.2%-100%), admission (*P*=.01), prescription of pharmacotherapy (*P*=.01), and deferral of surgery to the elective setting (*P*=.04). Although performance in perioperative medication management was consistent (*P*=.59), the LLMs erred in 15.0% to 29.5% of cases.

**Table 3.** Scores by disease.

Disease	Cases (n=42), n (%)	Overall score, mean (SD; range)	Extra suggestions (%)	Unsafe plans, n/N (%)	Word count, mean (SD)
ALI^a^	5 (11.9)	16.5 (2.7; 10-20)	28.6	8/15 (53.3)	913 (198)
Aortopathy	10 (23.8)	16.8 (2.9; 5-20)	66.7	1/30 (3.3)	626 (238)
CLI^b^	7 (16.7)	17.2 (2.1; 14-20)	76.2	2/21 (9.5)	891 (355)
DFI^c^	10 (23.8)	17.2 (1.9; 13-20)	60.0	2/30 (6.7)	801 (222)
ECD^d^	10 (23.8)	16.1 (1.3; 14-18)	63.3	4/30 (13.3)	755 (186)

a ALI: acute limb ischemia.

b CLI: chronic limb ischemia.

c DFI: diabetic foot infection.

d ECD: extracranial cerebrovascular disease.

### Predictors of Unsafe Plans

Univariable analysis identified lower overall score, longer word count, and ALI cases as significant predictors of unsafe plans (Table 4). In multivariable logistic regression, these 3 variables remained independently predictive of potential harm to patients (low overall score: OR 0.471, 95% CI 0.306‐0.722*; P*=.001; high word count: OR 1.003, 95% CI 1.001‐1.005*; P*=.001; and diagnosis of ALI: OR 20.9, 95% CI 3.79‐61.8*; P*<.001). Once adjusted for these factors, LLM identity was not associated with unsafe outputs. The association between word count and unsafe suggestions is shown in Supplementary Figure S1 in Multimedia Appendix 4.

**Table 4.** Regression analysis of factors predictive of unsafe suggestions.

Predictor	Univariable OR^a^ (95% CI)	Univariable *P* value	Multivariable OR (95% CI)	Multivariable *P* value
Composite score	0.47 (0.30‐0.74)	.001	0.471 (0.306‐0.722)	.001
Word count	1.003 (1.001-1.006)	.008	1.003 (1.001‐1.005)	.001
LLM^b^			—^c^	—
ChatGPT	1.68 (0.42‐6.75)	.46		
Gemini	0.79 (0.11‐5.76)	.81		
Claude	0.55 (0.09‐3.37)	.52		
Disease				
ALI^d^	22.3 (3.8‐51.1)	.001	20.9 (3.79‐61.8)	<.001
Aortopathy	0.03 (0.00‐2.67)	.12	—	—
CLI^e^	0.47 (0.09‐2.48)	.38	—	—
DFI^f^	1.28 (0.35‐4.66)	.71	—	—
ECD^g^	0.3 (0.04‐2.02)	.21	—	—

a OR: odds ratio.

b LLM: large language model.

c Not applicable.

d ALI: acute limb ischemia.

e CLI: chronic limb ischemia.

f DFI: diabetic foot infection.

g ECD: extracranial cerebrovascular disease.

## Discussion

### Principal Findings

The 3 LLMs (ChatGPT, Gemini, and Claude) responded to 42 vascular surgery cases with reasonable success, achieving mean composite scores of 86.5%, 83.5%, and 88.0%, respectively. All LLMs performed comparably, with no one model significantly outperforming the others. These results fit with the corpus of current literature, which finds that LLMs can obtain scores between 70% and 90% in free-response questions on various surgical subspecialties, including vascular surgery [16,17], general surgery [18], neurosurgery [19], and orthopedics [20]. Domain-specific assessment also demonstrates mostly consistent scores of 80% to 90% in aspects of workup and multimodal management by model. Because transformer models recognize associations learned from large datasets, LLMs can suggest additional management steps when relevant clinical triggers appear. In our cohort, they frequently proposed appropriate adjunctive actions such as drug and alcohol support for smokers, obstructive sleep apnea or bariatric surgery referral for patients with obesity, and coronary artery screening in patients presenting with CLI, highlighting their potential role as supportive tools in physician-led decision-making. Comparative studies have likened LLMs to junior registrars in their performance, with a study by Palenzuela et al [18] finding that LLMs significantly outperformed junior registrars in the management of general surgical pathologies (79.2% vs 66.2%; *P*=.009), with their performance being similar to that of senior trainees (76.0%) and consultants (77.6%). Furthermore, it is important to acknowledge that human clinicians too will err, make unsafe plans, and omit key details. Hence, building on the results of this study, a pilot study directly comparing clinician with LLM respondents will be useful to further define the role of LLMs in clinical practice.

However, this study also highlights several barriers to LLMs’ unsupervised integration into clinical practice. A key finding of this study is the distinction it draws between quality (overall score) and safety. Despite obtaining mean composite scores above 80% for all models and across all pathologies, substantial variability remained in the safety of the proposed management plans. While higher composite scores were independently protective against unsafe plans, other factors were involved, and a substantial proportion of high-scoring outputs still contained potentially dangerous recommendations. It is concerning that over half of all ALI plans were flagged as unsafe compared with single-digit to low-teens percentages in the other vascular conditions. On multivariable analysis, ALI diagnosis, lower overall management score, and higher word count were all independent predictors of unsafe plans.

The ALI subgroup comprised only 5 cases (15 model outputs), reflecting its real-world lower prevalence and narrower case mix. Although the OR for unsafe outputs in ALI was large, it should be interpreted cautiously and considered hypothesis generating rather than definitive, particularly given the wide CIs. Several ALI cases generated more than 1 unsafe recommendation (10 unsafe suggestions across 8 unsafe plans), suggesting a consistent safety signal. Despite ALI remaining independently associated with unsafe plans after adjustment for composite score and word count, given the small sample size, a small number of reclassifications could materially alter the magnitude of the association. Therefore, confirmation with larger, ALI-specific cohorts is required before drawing definitive conclusions. Moreover, the association between response length and unsafe outputs should not be interpreted as causal. Longer responses inherently contain more management elements, increasing the probability that at least one may be unsafe. Extended outputs may also reflect model uncertainty or greater clinical complexity, both of which could independently increase risk. Additionally, longer responses may expose models to a greater likelihood of confabulated or hallucinated recommendations.

The risk associated with LLM-derived management plans is detailed elsewhere in the literature, with a study of 200 short clinical vignettes in vascular surgery finding that, despite achieving 89% in a checklist-based marking score, ChatGPT missed key information in 17.5% of cases and generated responses with a moderate to high risk of harm in 13% of cases [16]. Additionally, a study of ChatGPT’s responses to fellowship-level multiparagraph written answer response assessments in otorhinolaryngology found that, despite the LLM scoring highly enough to achieve a passing grade, up to 33% of responses espoused invalid or conflicting information [21].

Although, as referenced above, the performance of LLMs in various fields has been studied extensively [13], the results of this study are indeed contributory to the field of vascular surgery. The transformer technology that makes LLMs adept at formulating responses also makes them vulnerable to confabulation: models are taught to generate text by finding the “next token” within the training data to formulate the “most likely” answer to a question. However, in cases in which training data contain conflicting or insufficient information, LLMs may produce incorrect content and create false word associations [22]. Given that deficiencies in training data can increase the risk of incorrect responses and neural hallucinations and that users in various fields are placing their trust in LLM outputs, there is an urgent need for their validation in each clinical specialty. No studies to date have thoroughly assessed the ability of LLMs to coordinate holistic care in vascular surgery cases. In real-world scenarios, clinical problems presented to LLMs will not be in the form of multiple-choice or true-or-false questions but in the format assessed in this study.

A further barrier to the trustworthiness of LLM responses is these models’ struggle to express uncertainty to users [23]. A 2025 study by Steyvers et al [24] involving 301 participants typified the phenomenon of “automation bias,” demonstrating that users place significant trust in LLMs due to their confidently worded responses—trust that grows proportionately to response length. However, our data draw a negative correlation between response length and safety, further highlighting the discrepancy between users’ perceptions and the reality of LLM outputs.

Another key finding is the significant and unpredictable variability identified within LLMs’ plans. Diagnostic accuracy was variable, with ALI being diagnosed correctly in 46.7% of cases, CLI diagnosed correctly in 76.2% of cases, and ECD diagnosed correctly in 80.0% of cases, whereas aortopathy and DFI were correctly identified in 100% of cases (*P*<.001; η^2^=0.24). Decision for admission, surgical timing, and prescription of pharmacotherapy all showed noteworthy variability between diseases, and nonpharmacological management and multidisciplinary team involvement in the different models also significantly varied (*P*<.05 in each case). Concerningly, management plans for ALI were consistently more unsafe than for any other pathology (53.3% vs 3.3%-16.7%; *P*<.005 in each instance). Cases of ALI are frequently time-critical, with patients often presenting in extremis, which could potentially expose a patient to risk in the hands of an inexperienced user (such as a junior on-call registrar). This variability has been widely reported in cases in which LLMs have been applied to clinical surgery. A recent European series found that ChatGPT scored 65% in 385 case-based multiple-choice questions in vascular surgery; however, there was marked variability between pathologies. Questions pertaining to portal hypertension and surgical complications were answered correctly in less than 50% of cases, despite the model scoring highly (≥75%) in cases of lymphedema and vascular access [25].

One promising strategy to improve LLM outputs is retrieval-augmented generation (RAG), where a user can include a database of choice containing verified external information (such as journal articles, textbooks, or clinical practice guidelines) with an LLM’s training data [26]. By providing only relevant content, the LLM’s responses become focused on a specific area, improving accuracy and reducing hallucinations. One such database is VASC.AI, a pool of more than 250,000 trials, guidelines, and abstracts in vascular surgery that can be used for RAG. A recent validation of this technique showed that it improved GPT-4’s accuracy in answering 244 questions from the fifth edition of the Vascular Education and Self-Assessment Program for American vascular trainees from 77.7% (SD 7.6%) to 93.8% (SD 2.4%; *P*<.001) across a range of vascular pathologies, including aortoiliac and peripheral artery disease, and cerebrovascular disease [27]. Rates of information error were lower with RAG; however, the logical error rate rose slightly when RAG was applied to GPT-4, showing that the models may struggle to integrate new information into their training data—underscoring the need for further refinement of this technology. Optimistic results have also been reported when RAG has been applied elsewhere, such as hepatology [28], urology [29], and endocrinology [30].

A further strategy for optimizing the specificity and relevance of LLM outputs is via the use of prompt engineering, which we used in this study by contextualizing each prompt with “You are a vascular surgeon...” As LLM performance is known to be sensitive to prompt structure, our use of a structured, role-assigned prompt with predefined questions likely represents an optimized use case scenario. Prior digital health studies have demonstrated that prompt engineering techniques can improve factual alignment and reduce unsafe outputs but also highlight variability with small changes in wording [31]. Accordingly, our findings reflect model performance under controlled prompting conditions and may not generalize to unstructured use, underscoring another risk of LLM use in clinical scenarios by an untrained user.

Before LLMs can be used freely in clinical practice, their ethical and legal concerns must be addressed. Data privacy remains an ongoing issue, especially where health data are concerned. For example, OpenAI states the following in its privacy policy: “We collect Personal Data that you provide in the input to our Services (‘Content’), including your prompts and other content you upload,” which may be shared with “vendors...service providers...government authorities or other third parties” [32]. Accordingly, the use of real health data on such platforms would raise ethical concerns; therefore, fictional cases were used in this study. In Australia, under the Privacy Act 1988 and Australian Privacy Principles, users must obtain explicit patient consent, highlighting the risks of data reidentification and distribution, prior to uploading sensitive health information to overseas databases, although specific regulation of LLMs in Australia is currently in its infancy [33]. In addition to privacy concerns, LLMs are not currently licensed by the Therapeutic Goods Administration as medical devices in Australia and, hence, cannot be deployed as autonomous clinical tools. These and other risks have been outlined in a Royal Australasian College of Surgeons position statement on the safe and responsible integration of AI in Australian health care [34]. Given the rapid pace of technological advancement in this space, the Royal Australasian College of Surgeons advocates for updates to relevant laws such as the Privacy Act 1988, the Therapeutic Goods Act 1989, and the Health Practitioner Regulation National Law 2009 and for a national body governing AI in health care to oversee privacy, data security, error reporting, and clinical validation.

Concerns surrounding the use of LLMs in clinical surgery are not limited to Australia. A recent European survey of 342 vascular specialists revealed that, despite 81% of respondents believing that LLMs have the future potential to be beneficial to patient care, only 27% currently trusted LLM-generated clinical information. Issues such as variability, inaccuracy, and inapplicability of outputs were noted by 74% of respondents, and 62% believed that these tools may in fact be harmful to patients in their current iterations [35].

Our findings argue against the uncritical use of LLM outputs in vascular surgery. The absence of major performance differences among the 3 models suggests that the choice of LLM may matter less than how the model is used, how its outputs are framed, and what governance surrounds it. Future vascular-specific models, particularly those incorporating local guidelines and registry data via RAG, may meaningfully shift this balance but will require equally rigorous evaluation.

Strengths of this study include the use of complex, consultant-derived case vignettes spanning the spectrum of vascular practice; a detailed scoring rubric covering diagnosis, investigation, surgical and medical management, and consults; and a separate assessment of safety. Each model responded independently to identical prompts, permitting direct comparison under controlled conditions. However, several key limitations must be acknowledged:

- Our gold standard was a single vascular unit’s consensus view, which may not capture all reasonable practice patterns or international variation.
- Each response was discussed and graded by a panel against a set standard rather than raters independently scoring each output. As a result, interrater reliability statistics were not calculated, introducing group-level bias and limiting reproducibility.
- Models and training data are evolving rapidly; our results represent a snapshot of LLM performance and iteration at a specific time.
- Each case was only given to each LLM once; hence, variability in performance within the same case could not be assessed. This is a limitation not only of this study but also of LLMs themselves given that, on repeated interrogation, LLM accuracy will vary.
- The sample size was small, especially in cases of ALI and CLI.
- A binary safety score was implemented in analyses; however, with a larger sample size of clinical cases, a severity rating scale for unsafe recommendations may be more informative.

Future research should prioritize prospective validation of LLMs against vascular surgeons in real-world clinical settings. Moreover, given that ideal conditions with strict prompt engineering were used to generate each LLM output, further study of the degree to which prompt engineering affects output accuracy and safety could be conducted, as this may better reflect actuarial LLM use. The equity of LLMs in managing diverse patient populations—particularly Indigenous and rural or remote Australians—also requires assessment to mitigate algorithmic biases that may exacerbate health care disparities. Additionally, research should focus on developing transparent LLMs with inbuilt error reporting systems compliant with Therapeutic Goods Administration regulations by revealing the reasoning and training data behind diagnostic and management suggestions.

### Conclusions

Despite their unprecedented ability to rapidly generate succinct, tailored, and largely accurate responses to clinical vascular surgery cases, LLMs cannot yet be safely deployed as clinical tools. Their propensity to generate unsafe and variable recommendations, the lack of a regulatory framework, and their ethical vulnerabilities impede their utility as everyday clinical companions.

## Supplementary material

10.2196/91973 Multimedia Appendix 1 Example clinical case.

10.2196/91973 Multimedia Appendix 2 List of textbooks and clinical practice guidelines used in the development of the marking rubric (alphabetical order).

10.2196/91973 Multimedia Appendix 3 Mean composite scores: (A) pooled composite score by model and (B) pooled composite score by disease.

10.2196/91973 Multimedia Appendix 4 Comparisons, breakdowns, and performance figures and tables of large language models for individual disease categories.

10.2196/91973 Multimedia Appendix 5 Unsafe recommendations by (A) model and (B) disease.

## Abbreviations

ALI: acute limb ischemia
CLI: chronic limb ischemia
DFI: diabetic foot infection
ECD: extracranial cerebrovascular disease
LLM: large language model
OR: odds ratio
RAG: retrieval-augmented generation
