# Supplementary material for: Evaluating the Performance of Large Language Models in Vascular Surgery Cases: Cross-Sectional Study
Source: JMIR Perioper Med. 2026 Nov 6;9:e91973. doi: 10.2196/91973 (PMC13743064; doi:10.2196/91973)
Supplement: Supplementary file 1 [file periop-v9-e91973-s001.docx]

**Appendix 1**

Example Clinical Case – diabetic foot infection

Age: 59

Sex: Male

History of presenting complaint:

- One week severe pain in the medial right forefoot
- Worsening malaise and fevers
- Eight weeks ago underwent 2^nd^ and 3^rd^ ray amputation, with wounds not healing despite negative pressure wound dressings
- Presented due to concern from community nurses about possible wound infection.

Past medical history:

- Ischaemic heart disease, previous right coronary artery stent.
- Ischaemic cardiomyopathy with heart failure – left ventricular ejection fracture 22%
- Hypertension
- Dyslipidaemia
- Insulin-dependent type 2 diabetes mellitus
- Gout

Regular medications

- Aspirin 100mg/day
- Clopidogrel 75mg/day
- Insulin aspart 10U TDS with meals
- Insulin glargine 12U BD
- Losartan 50mg/day
- Rosuvastatin 20mg/day
- Bisoprolol 2.5mg/day
- Febuxostat 40mg/day

Past surgical history

- Left distal radius open reduction + internal fixation (30 years ago)
- Coronary angiogram / stenting (2 years ago)
- Right 2^nd^ and 3^rd^ ray amputations (8 weeks ago)

Allergies: nil.

Social history

- From home with family
- Independent
- Recently retired local government politician
- Smoking/drinking: never

Vital signs on admission

- Afebrile
- Normal heart rate and respiratory rate
- Borderline hypotension – chronic

Examination findings:

- Well from end of bed.
- Non healing wounds with necrotic tissue overlying cavity.
- Sinus tract on plantar aspect of wound probing to calcaneus expressing minimal pus.
- Wet necrotic skin on the plantar surface of the foot with peri-wound cellulitis with no tracking lymphangitis.
- Palpable dorsalis pedis and posterior tibialis pulses.
- Neurologically in-tact.
- No wounds on left foot.
- Toe pressures: right foot 99mmHg, left foot 79mmHg.

Blood results

- Haemoglobin 103 g/L
- White cell count 17.9 x10^9^/L
- Estimated GFR >90 mg/min/1.73m^2^
- CRP 108 mg/L
- Lactate 0.9 mmol/L
- HbA1c 11.1%
- Random blood sugar level 6.2 mmol/L
- Sodium 129mmol/L
