# Supplementary material for: Evaluating the Performance of Large Language Models in Vascular Surgery Cases: Cross-Sectional Study
Source: JMIR Perioper Med. 2026 Nov 6;9:e91973. doi: 10.2196/91973 (PMC13743064; doi:10.2196/91973)
Supplement: Supplementary file 2 [file periop-v9-e91973-s002.docx]

Appendix 2 – Compilation of textbooks and clinical practice guidelines used in the development of the marking rubric (alphabetical order)

AbuRahma AF, Avgerinos ED, Chang RW, et al. Society for Vascular Surgery clinical practice guidelines for management of extracranial cerebrovascular disease. J Vasc Surg. 2022 Jan;75(1S):4S-22S.

Björck M, Earnshaw JJ, Acosta S, et al. Editor's Choice - European Society for Vascular Surgery (ESVS) 2020 Clinical Practice Guidelines on the Management of Acute Limb Ischaemia. Eur J Vasc Endovasc Surg. 2020 Feb;59(2):173-218

Conte MS, Bradbury AW, Kolh P,et al. Global Vascular Guidelines on the Management of Chronic Limb-Threatening Ischemia. Eur J Vasc Endovasc Surg. 2019 Jul;58(1S):S1-S109.e33.

Farber A, Angle N, Avgerinos E, et al. The Society for Vascular Surgery clinical practice guidelines on popliteal artery aneurysms. J Vasc Surg. 2022 Jan;75(1S):109S-120S

Naylor R, Rantner B, Ancetti S, et al. Editor's Choice - European Society for Vascular Surgery (ESVS) 2023 Clinical Practice Guidelines on the Management of Atherosclerotic Carotid and Vertebral Artery Disease. Eur J Vasc Endovasc Surg. 2023 Jan;65(1):7-111.

Nelson MR, Banks E, Brown A, et al. 2023 Australian guideline for assessing and managing cardiovascular disease risk. Med J Aust. 2024 May 20;220(9):482-490

Nordanstig J, Behrendt CA, Baumgartner I, et al. Editor's Choice -- European Society for Vascular Surgery (ESVS) 2024 Clinical Practice Guidelines on the Management of Asymptomatic Lower Limb Peripheral Arterial Disease and Intermittent Claudication. Eur J Vasc Endovasc Surg. 2024 Jan;67(1):9-96

Schaper NC, van Netten JJ, Apelqvist J, et al. Practical guidelines on the prevention and management of diabetes-related foot disease (IWGDF 2023 update). Diabetes Metab Res Rev. 2024 Mar;40(3):e3657

Sidawy, A. N., & Perler, B. A. (2022). Rutherford's Vascular Surgery and Endovascular Therapy, Tenth Edition: Volume 1-2. Elsevier.

Wanhainen A, Van Herzeele I, Bastos Goncalves F, et al. Editor's Choice -- European Society for Vascular Surgery (ESVS) 2024 Clinical Practice Guidelines on the Management of Abdominal Aorto-Iliac Artery Aneurysms. Eur J Vasc Endovasc Surg. 2024 Feb;67(2):192-331.
