# Supplementary material for: Evaluating the Performance of Large Language Models in Vascular Surgery Cases: Cross-Sectional Study
Source: JMIR Perioper Med. 2026 Nov 6;9:e91973. doi: 10.2196/91973 (PMC13743064; doi:10.2196/91973)
Supplement: Supplementary file 3 [file periop-v9-e91973-s004.docx]

Supplementary Figure S1: Distribution of safe and unsafe suggestions by word count.

Supplementary Table S1 – Comparison of overall score, word count, propensity to suggest extra management options and unsafe suggestions between models. SD = standard deviation.

| **Comparison** | **Overall Score (% mean ± SD)** | **Word Count (mean ± SD)** | **Extra Suggestions (%)** | **Unsafe Plans (%)** |
| --- | --- | --- | --- | --- |
| **ChatGPT vs Gemini** | 85.7 ± 10.1 vs 83.4 ± 12.3 (p = 0.24) | **664 ± 134 vs 951 ± 316 (p = 0.033)** | 56% vs 62% (p = 0.39) | 11.7% vs 24.2% (p = 0.077) |
| **ChatGPT vs Claude** | 85.7 ± 10.1 vs 87.2 ± 9.8 (p = 0.47) | 664 ± 134 vs 715 ± 199 (p = 0.42) | 56% vs 71% (p = 0.091) | 11.7% vs 7.5% (p = 0.36) |
| **Claude vs Gemini** | 87.2 ± 9.8 vs 83.4 ± 12.3 (p = 0.16) | **715 ± 199 vs 951 ± 316 (p = 0.041)** | 71% vs 62% (p = 0.47) | 7.5% vs 24.2% (p = 0.062) |

Supplementary Table S2 – Breakdown of unsafe suggestions into the five pre-determined subcategories: (1) Diagnostic error or delay; (2) Delayed or inappropriate surgical intervention; (3) Inappropriate prescription or withholding of pharmacotherapy; (4) Inappropriate adjunctive therapy; (5) Unsafe disposition. Ao=aortopathy. ALI=acute limb ischaemia. CLI=chronic limb ischaemia. DFI=diabetic foot infection. ECD=extracranial cerebrovascular disease.

| Disease | Unsafe Plans (%) |  |
| --- | --- | --- |
| ALI | 8 (53.3) | - *Diagnostic error or delay =0* - *Delayed or inappropriate surgical intervention =3* - *Inappropriate prescription or withholding of pharmacotherapy =1* - *Inappropriate adjunctive therapy =6* - *Unsafe disposition =0* |
| Ao | 1 (3.3) | - *Diagnostic error or delay =0* - *Delayed or inappropriate surgical intervention =0* - *Inappropriate prescription or withholding of pharmacotherapy =1* - *Inappropriate adjunctive therapy =0* - *Unsafe disposition =0* |
| CLI | 2 (9.5) | - *Diagnostic error or delay =0* - *Delayed or inappropriate surgical intervention =0* - *Inappropriate prescription or withholding of pharmacotherapy =0* - *Inappropriate adjunctive therapy =1* - *Unsafe disposition =1* |
| DFI | 2 (6.6) | - *Diagnostic error or delay =1* - *Delayed or inappropriate surgical intervention =1* - *Inappropriate prescription or withholding of pharmacotherapy =0* - *Inappropriate adjunctive therapy =0* - *Unsafe disposition =1* |
| ECD | 4 (13.3) | - *Diagnostic error or delay =0* - *Delayed or inappropriate surgical intervention =1* - *Inappropriate prescription or withholding of pharmacotherapy =4* - *Inappropriate adjunctive therapy =0* - *Unsafe disposition =1* |

Supplementary Table S3 – pairwise comparison of the performance of each large language model by disease. Ao=aortopathy. ALI=acute limb ischaemia. CLI=chronic limb ischaemia. DFI=diabetic foot infection. ECD=extracranial cerebrovascular disease.

| **Disease** | **Model 1** | **Model 1 Mean ± SD** | **Model 2** | **Model 2 Mean ± SD** | **p-value** |
| --- | --- | --- | --- | --- | --- |
| ALI | **ChatGPT** | 16.4 ± 2.7 | **Gemini** | 15.0 ± 3.1 | 0.4669 |
| ALI | **ChatGPT** | 16.4 ± 2.7 | **Claude** | 18.2 ± 1.8 | 0.2494 |
| **ALI** | **Gemini** | **15.0 ± 3.1** | **Claude** | **18.2 ± 1.8** | **0.0395** |
| Ao | **ChatGPT** | 16.8 ± 2.0 | **Gemini** | 16.2 ± 4.1 | 0.6863 |
| Ao | **ChatGPT** | 16.8 ± 2.0 | **Claude** | 17.2 ± 2.1 | 0.6969 |
| Ao | **Gemini** | 16.2 ± 4.1 | **Claude** | 17.2 ± 2.1 | 0.5189 |
| CLI | **ChatGPT** | 17.8 ± 2.5 | **Gemini** | 17.2 ± 2.2 | 0.6564 |
| CLI | **ChatGPT** | 17.8 ± 2.5 | **Claude** | 16.5 ± 1.6 | 0.272 |
| CLI | **Gemini** | 17.2 ± 2.2 | **Claude** | 16.5 ± 1.6 | 0.5074 |
| DFI | **ChatGPT** | 17.1 ± 1.7 | **Gemini** | 16.8 ± 2.4 | 0.7528 |
| DFI | **ChatGPT** | 17.1 ± 1.7 | **Claude** | 17.7 ± 1.7 | 0.4957 |
| DFI | **Gemini** | 16.8 ± 2.4 | **Claude** | 17.7 ± 1.7 | 0.3834 |
| **ECD** | **ChatGPT** | **16.3 ± 0.7** | **Gemini** | **15.4 ± 1.3** | **0.0457** |
| ECD | **ChatGPT** | 16.3 ± 0.7 | **Claude** | 16.7 ± 1.4 | 0.3711 |
| **ECD** | **Gemini** | **15.4 ± 1.3** | **Claude** | **16.7 ± 1.4** | **0.0346** |

Supplementary Table S4 – Breakdown of Composite Scores by Model

| **Domain** | **ChatGPT (%)** | **Gemini (%)** | **Claude (%)** | **F** | **p-value** | **η²** |
| --- | --- | --- | --- | --- | --- | --- |
| Diagnosis | 78.6 | 92.9 | 83.3 | 1.74 | 0.18 | 0.03 |
| Investigation | 87.1 | 81.3 | 87.0 | 0.89 | 0.41 | 0 |
| Admission | 92.9 | 88.1 | 90.5 | 0.24 | 0.78 | 0 |
| Urgent Surgery | 97.6 | 92.9 | 97.6 | 0.36 | 0.7 | 0.01 |
| Elective Surgery | 90.5 | 83.3 | 88.1 | 0.49 | 0.61 | 0.01 |
| Correct Surgery | 88.1 | 78.6 | 90.5 | 1.36 | 0.26 | 0.02 |
| Pharmacotherapy | 80.4 | 80.6 | 79.9 | 0.01 | 0.99 | 0 |
| Perioperative Medication Management | 77.8 | 73.5 | 80.9 | 0.43 | 0.65 | 0 |
| Non-Pharmacological Management | 85.7 | 81.0 | 90.5 | **2.06** | **0.013** | **0.06** |
| Consults | 89.7 | 83.3 | 92.9 | **3.16** | **0.045** | **0.06** |

Supplementary Table S5 – Breakdown of Composite Scores by Disease. Ao=aortopathy. ALI=acute limb ischaemia. CLI=chronic limb ischaemia. DFI=diabetic foot infection. ECD=extracranial cerebrovascular disease.

| **Domain** | **ALI (%)** | **Ao (%)** | **CLI (%)** | **DFI (%)** | **ECD (%)** | **F** | **p-value** | **η²** |
| --- | --- | --- | --- | --- | --- | --- | --- | --- |
| **Diagnosis** | **46.7** | **100** | **76.2** | **100** | **80.0** | **9.29** | **<0.001** | **0.24** |
| Investigation | 75.6 | 86.7 | 81.0 | 90.0 | 86.4 | 1.31 | 0.27 | 0 |
| **Admission** | **100** | **83.3** | **100** | **90.0** | **80.0** | **3.91** | **0.010** | **0.11** |
| Urgent Surgery | 100 | 96.7 | 100 | 93.3 | 86.7 | 2.32 | 0.061 | 0.07 |
| **Elective Surgery** | **80.0** | **93.3** | **90.5** | **96.7** | **73.3** | **2.48** | **0.047** | **0.08** |
| Correct Surgery | 80.0 | 93.3 | 76.2 | 93.3 | 80.0 | 1.41 | 0.24 | 0.04 |
| **Pharmacotherapy** | **80.0** | **72.2** | **84.1** | **74.4** | **91.7** | **3.25** | **0.014** | **0.10** |
| Perioperative Medication Management | 83.3 | 73.5 | 85.0 | 70.5 | 76.7 | 0.7 | 0.59 | 0 |
| Non-Pharmacological Management | 73.3 | 84.4 | 90.5 | 88.9 | 86.7 | 1.72 | 0.15 | 0.05 |
| Consults | 93.3 | 93.3 | 79.4 | 90.0 | 86.7 | 2.4 | 0.051 | 0.07 |

Supplementary Table S6: Pairwise comparison by disease, pooled across large language models’ responses. Composite scores compared by t-testing, safety and extra suggestions compared via chi-squared analysis. Ao=aortopathy. ALI=acute limb ischaemia. CLI=chronic limb ischaemia. DFI=diabetic foot infection. ECD=extracranial cerebrovascular disease.

| Disease 1 | Disease 2 | Disease 1 Score  Mean ± SD | Disease 2 Score  Mean ± SD | Score p-value | Disease 1 Safety (%) | Disease 2 Safety (%) | Safety p-value | Extra Suggestion 1 | Extra Suggestion 2 | Extra Suggestion p-value |
| --- | --- | --- | --- | --- | --- | --- | --- | --- | --- | --- |
| **Ao** | **DFI** | 16.76 ± 2.86 | 17.21 ± 1.94 | 0.473 | 3.3% | 6.7% | 0.561 | 66.7% | 60.0% | 0.789 |
| **Ao** | **ALI** | 16.76 ± 2.86 | 16.53 ± 2.75 | 0.805 | **3.3%** | **53.3%** | **<0.0001** | **66.7%** | **28.6%** | **0.032** |
| **Ao** | **CLI** | 16.76 ± 2.86 | 17.19 ± 2.10 | 0.555 | 3.3% | 9.5% | 0.365 | 66.7% | 76.2% | 0.673 |
| **Ao** | **ECD** | 16.76 ± 2.86 | 16.14 ± 1.26 | 0.284 | 3.3% | 16.7% | 0.088 | 66.7% | 63.3% | 1.000 |
| **DFI** | **ALI** | 17.21 ± 1.94 | 16.53 ± 2.75 | 0.343 | **6.7%** | **53.3%** | **0.0002** | 60.0% | 28.6% | 0.106 |
| **DFI** | **CLI** | 17.21 ± 1.94 | 17.19 ± 2.10 | 0.971 | 6.7% | 9.5% | 0.716 | 60.0% | 76.2% | 0.236 |
| **DFI** | **ECD** | **17.21 ± 1.94** | **16.14 ± 1.26** | **0.014** | 6.7% | 16.7% | 0.235 | 60.0% | 63.3% | 1.000 |
| **ALI** | **CLI** | 16.53 ± 2.75 | 17.19 ± 2.10 | 0.421 | **53.3%** | **9.5%** | **0.0029** | **28.6%** | **76.2%** | **0.015** |
| **ALI** | **ECD** | 16.53 ± 2.75 | 16.14 ± 1.26 | 0.510 | **53.3%** | **16.7%** | **0.0097** | **28.6%** | **63.3%** | **0.048** |
| **CLI** | **ECD** | **17.19 ± 2.10** | **16.14 ± 1.26** | **0.029** | 9.5% | 16.7% | 0.476 | 76.2% | 63.3% | 0.344 |
